# Insights into the proteomic profile and gene expression of *Lutzomyia longipalpis*-derived Lulo cell line

**DOI:** 10.1590/0074-02760200113

**Published:** 2020-10-26

**Authors:** Luzia Monteiro de Castro Côrtes, Daniela de Pita-Pereira, Priscila Silva Grijó Farani, Bernardo Acácio Santini Pereira, Geovane Dias-Lopes, Franklin Souza da Silva, Paloma Resende Corrêa, Roger Magno Macedo Silva, Suzana Côrte-Real, Felio Jesus Bello, Leila Mendonça-Lima, Otacilio da Cruz Moreira, Mariana Caldas Waghabi, Carlos Roberto Alves

**Affiliations:** 1 Fundação Oswaldo Cruz-Fiocruz, Instituto Oswaldo Cruz, Laboratório de Biologia Molecular e Doenças Endêmicas, Rio de Janeiro, RJ, Brasil; 2 Fundação Oswaldo Cruz-Fiocruz, Instituto Oswaldo Cruz, Laboratório de Genômica Funcional e Bioinformática, Rio de Janeiro, RJ, Brasil; 3 Fundação Oswaldo Cruz-Fiocruz, Instituto Oswaldo Cruz, Plataforma de Microscopia Eletrônica Rudolf Barth, Rio de Janeiro, RJ, Brasil; 4 Fundação Oswaldo Cruz-Fiocruz, Instituto Oswaldo Cruz, Laboratório de Biologia Estrutural, Rio de Janeiro, RJ, Brasil; 5 Facultad de Ciencias Agropecuarias, Programa de Medicina Veterinaria, Universidad de La Salle, Bogotá, Colombia

**Keywords:** Lulo cell, Leishmania (Viannia) braziliensis, proteomic, RT-qPCR

## Abstract

**BACKGROUND:**

*Lutzomyia longipalpis*-derived cell line (Lulo) has been suggested as a model for studies of interaction between sandflies and *Leishmania*.

**OBJECTIVES:**

Here, we present data of proteomic and gene expression analyses of Lulo cell related to interactions with *Leishmania (Viannia) braziliensis*.

**METHODS:**

Lulo cell protein extracts were analysed through a combination of two-dimensional gel electrophoresis and mass spectrometry and resulting spots were further investigated *in silico* to identify proteins using Mascot search and, afterwards, resulting sequences were applied for analysis with VectorBase.

**RESULTS:**

Sixty-four spots were identified showing similarities to other proteins registered in the databases and could be classified according to their biological function, such as ion-binding proteins (23%), proteases (14%), cytoskeletal proteins (11%) and interactive membrane proteins (9.5%). Effects of interaction with *L. (V.) braziliensis* with the expression of three genes (enolase, tubulin and vacuolar transport protein) were observed after an eight-hour timeframe and compared to culture without parasites, and demonstrated the impact of parasite interaction with the expression of the following genes: LLOJ000219 (1.69-fold), LLOJ000326 (1.43-fold) and LLOJ006663 (2.41-fold).

**CONCLUSIONS:**

This set of results adds relevant information regarding the usefulness of the Lulo cell line for studies with *Leishmania* parasites that indicate variations of protein expression.

Leishmaniases are parasitic diseases whose etiological agents are protozoa of the genus *Leishmania* that are transmitted by insect vectors and can occur in the cutaneous form [cutaneous leishmaniasis (CL)] and in the visceral form [visceral leishmaniasis (VL)]. The parasites are transmitted by the bite of female insects of the genus *Phlebotomus* (Diptera: Psychodidae: Phlebotominae), in the Old World, and of the genus *Lutzomyia* in the New World.^1^ ^,^ ^2^ Due to the high incidence rate and the capacity to produce deformities and even deaths, leishmaniases are described as diseases of interest to public health. These diseases are included in the group of neglected tropical diseases (NTDs) due to the lack of investment by the pharmaceutical industry, current unavailability of effective or adequate treatments and association with poor populations. NTDs contribute to maintaining the structure of social inequality, as they may represent a strong obstacle to the development of affected populations.^3^ ^,^ ^4^

The complex network of leishmaniases transmission includes different species of parasites, vectors and reservoirs, where approximately 20 different *Leishmania* spp. pathogenic to humans are transmitted by an array of sand fly species. The parasite *Leishmania (Viannia) braziliensis* is the most prevalent etiological agent of American CL (ACL) throughout various countries in Central and South America and may exhibit several clinical forms, including the typical cutaneous form, which is the most common presentation, disseminated by atypical cutaneous (lupoid and verrucous) and mucocutaneous.^5^ Additionally, in endemic areas of ACL in Brazil, the transmission of *L*. (*V*.) *braziliensis* is driven by vectors such as *Lutzomyia (Psychodopygus) wellcomei, Lutzomyia (Nyssomyia) whitmani, Lutzomyia (Psychodopygus) davisi and Lutzomyia (Nyssomyia) intermedia* and it is directly related to vectorial capacity of these species.^6^ ^-^ ^9^ ^)^ Likewise, the geographic expansion of ACL in recent decades, which involves a diversity of *Leishmania* spp. transmitted by different sand fly species, has been associated with environmental and climatic changes in different regions, including peri-urban areas in certain cities in Brazil.^10^ ^-^ ^12^

The transmission of these parasites by sandflies is essential in maintaining the disease cycle and at least two factors determine the vectorial capacity for *leishmaniasis* transmission: the ability to colonise human-changed environments and the competence for feeding on human blood. Furthermore, there are species of sandflies that are vectors for only one *Leishmania* sp., whereas others can serve as hosts for multiple species. These latter species are classified as “permissible vectors”^13^ and include, for example, *Lu. Longipalpis*,^14^ *Phlebotomus argentipes*,^15^ ^)^ *Phlebotomus halepensis* ^16^ ^)^ and *Phlebotomus arabicus*.^17^

The mechanisms that regulate this species-specificity are not fully understood, and various components of the parasite may be mediators of this process, such as the flagellum protein of the promastigote.^18^ Moreover, there is a large diversity of ligands and receptors that are species-specific, mediating parasite-phlebotomine interactions that are not yet fully known.^19^ ^,^ ^20^ In regard to *Lu. longipalpis*, which is *Leishmania infantum* vector, studies on experimental infections support the development of other *Leishmania* spp., including *L. (V.) braziliensis*.^20^

In this context, the sequencing of the phlebotomine genome has been pointed out as a necessary and important advance to access the sandfly ligands or receptors related to vectorial capacity. Complete genomes are currently available only for *Phlebotomus papatasi* and *Lu. longipalpis* species.^21^ These species were chosen for several reasons: (i) both are disease vectors with wide geographical distribution - *Lu. longipalpis* is found from Mexico to Argentina,^22^ while *P. papatasi* is present in most of the Indian subcontinent, Middle East and Mediterranean sub-region; (ii) each species shares a high similarity with other species from the same genus - for example, salivary transcripts of *P. papatasi* were compared with different species of *Phlebotomus* (*Phlebotomus ariasi, Phlebotomus perniciousus* and *Phlebotomus duboscqi*) and the genotype revealed similarity with *Lu. longipalpis* and homology with *Lu. intermedia*;^19^ and (iii) each of these species transmits parasites at opposite ends of the pathological spectrum - *P. papatasi* transmits species associated with CL and *Lu. longipalpis* transmits only one specie associated with VL.^20^ It is important to note that in addition to the complete genome, the midgut transcriptome for both species has also been extensively analysed, adding further genomic data.^23^ Moreover, a large set of cDNA transcripts from the whole body of *Lu. longipalpis* has been sequenced, providing more information on gene expression features in sandflies.^23^

Notwithstanding the accumulated knowledge on genomes and transcriptomes, there are still few studies about the interaction and proliferation of *Leishmania* parasites in insect cell cultures. A first study demonstrated and confirmed the adhesion and proliferation of *Leishmania (L.) donovani* amastigotes in *Aedes albopictus* cells.^24^ Cells from *Aedes aegypti* were also reported as being susceptible to infection by *L. (L.) infantum*, *Leishmania (V.) braziliensis* ^25^ and *Leishmania (V.) panamensis*.^26^

Lulo cell line was previously established and characterised, showing susceptibility to infections by arboviruses, such as Mayaro, Ilheus, Changuinola, phlebovirus and by vesicular stomatitis viruses, as well as by *L. (L.) infantum*.^27^ ^,^ ^28^ Similarities between the infection process by *L. (L.) infantum* in Lulo cells and in the murine macrophage cell line J774 were also observed.^28^ Previously studies have reported that Lulo cells may be an adequate model for studying *Leishmania* spp. adhesion to its invertebrate host, as it was noted that species from both *Leishmania* subgenera show potential to adhere to Lulo cells. The species with higher adherence rates were *L. (L.) infantum, Leishmania (L.) amazonensis* and *L. (V.) braziliensis,* and adhesion of these parasites to Lulo cells can occur either by the parasite body or by its flagellum,^29^ which can be intermediated by glycosaminoglycans such as heparan sulphate on the Lulo cell surface.^30^

In addition, the Lulo cells were applied as a model to study the responses of the innate immune system of *L. (L.) infantum* in phlebotomines and it was observed that when bacteria *Wolbachia pipientis* were introduced in the Lulo cell cultures there was a decrease in the expression of genes related to the regulation of the Imd, Toll and Jak-Stat immune pathways, suggesting an immune activation. However, no negative effect of these bacteria on the *L. (L.) infantum* promastigotes infection of Lulo cell was observed *in vitro* assays.^31^ ^)^ Also, an efficient replication of arboviruses (dengue, yellow fever and chikungunya) was observed in the Lulo cells, reinforcing their importance in research on vector-pathogen interaction.^32^

Although the Lulo cells model was initially proposed to explore the events of adhesion with *L. (L.) infantum*, we have evidence that this cell can be useful in studies of adhesion for other *Leishmania* spp.^29^ In this context, the Lulo cell line can mimic the molecular events induced by *Leishmania* spp. in the intestine of the phlebotomine insect. The present study aimed to add new data on the Lulo cell, focusing specifically on its proteomic profile. The expression of certain genes (enolase, tubulin and vacuolar transport protein) from the Lulo cell during the adhesion of *L. (V.) braziliensis* promastigotes were also evaluated.

## MATERIALS AND METHODS

*Chemicals and reagents -* Sodium dodecyl sulfate (SDS), Tween 20, dithiothreitol (DTT), antibiotics (penicillin and streptomycin), radio-immunoprecipitation assay (RIPA) and protease inhibitor cocktail (cat #539134) were purchased from Calbiochem. Foetal bovine serum (FBS) was purchased from Cultilab S/A (Brazil). Coomassie Brilliant Blue G-250, acetonitrile (ACN), trichloroacetic acid (TCA), tributylphosphine (TBP), ampholytes and nonlinear immobilised pH gradient strips pH 3-10 (cat#1631112) were purchased from Bio-Rad Laboratories Inc. (USA). C18 ZipTip was purchased from Millipore-Applied Biosystems (Billerica Inc., USA). Trypsin Gold Mass Spectrometry Grade was purchased from PROMEGA (USA). TRIzol^®^ Reagent, Deoxyribonuclease I (DNase I) Amplification grade, SuperScript^®^ III First-Strand Synthesis System for RT-PCR and Qubit ssDNA Assay Kit were purchased from Invitrogen, Life Technologies, USA. SYBR Green PCR Master Mix was purchased from Applied Biosystems, Life Technologies, USA.

*Lulo cell line -* The Lulo cell line, obtained from embryonic tissue of *Lu*. *longipalpis,* was cultured as described previously,^27^ in a 1:1 mix of L15^33^ and Grace medium^34^ supplemented with 10% (v/v) FBS, penicillin (100 U/mL) and streptomycin (100 μg/mL) and incubated at 27ºC. After 48 h, 2 × 10^5^ cells/mL were washed twice with PBS for protein extraction. Additionally, these cells were stained with Giemsa to be observed with optical microscopy and processed for analysis by scanning electron microscopy.^29^

*Preparation of Lulo cell for proteome profile -* Lulo cells cultures were washed three times in PBS pH 7.2, harvested and re-suspended in RIPA buffer (cat # R0278) containing 1:100 protease inhibitor cocktail to obtain whole cell extract, which was used in the proteomic studies. The total Lulo cell proteins were precipitated in 17% (w/v) TCA followed by centrifugation (16,100 *g*, 5 min, 4°C) and the obtained pellet washed in cold acetone/triethanolamine 1% (v/v) in cold acetone. The pellet was then re-suspended in rehydration buffer (8 M urea, 2% (w/v) CHAPS, 4 mM TBP, 0.4% ampholytes pH 3-10) for isoelectric focusing (IEF). Protein concentration was determined by RCDC method (Bio Rad Laboratories, USA), using bovine serum albumin (BSA) as standard.

*Two-dimensional gel electrophoresis (2-DE) -* Nonlinear immobilised pH gradient (IPG) pH 3-10 strips were rehydrated (16 h, 25°C) in buffer (8 M urea, 2 mM tributhylphosphine, 1% ampholytes; w/v) containing 500 µg (17 cm) or 200 µg (7 cm) of Lulo cell protein extract. Isoelectric focusing was conducted on a Protean IEF Cell (Bio Rad) according to the manufacturer’s instructions. The strips were then re-equilibrated with 130 mM DTT and 135 mM iodoacetamide in equilibration buffer (6 M urea, 20% glycerol, 2% SDS), in sequence, for 15 min each. Proteins within the equilibrated strips were submitted to sodium dodecyl sulfate polyacrylamide gel electrophoresis (SDS-PAGE) in 12% gels.^35^ Proteins in gels were stained with colloidal Coomassie Brilliant Blue G-250, adapted from Neuhoff et al.^36^

*Image analysis -* Stained protein spots present in the gels were documented using a GS800 scanner (Bio Rad Laboratories, USA) and the images were analysed using the PDQuest software version 8.0.1 (Bio Rad Laboratories, USA). The spots were quantified based on their relative volume and the amount of protein in one spot was expressed as the sum of the intensities of all constituent pixels of that spot.^37^

*Protein digestion and tandem mass spectrometry (MS/MS) analysis -* Selected protein spots were manually excised from 17 cm gels and placed in 0.5 mL microtubes. Protein digestion and peptide extraction were conducted as previously described.^38^ Briefly, protein spots were excised, and the gel pieces washed three times with 50% (v/v) ACN in 25 mM ammonium bicarbonate for 15 min each, dehydrated in ACN and dried in Speed Vac SC110 (refrigerated vapour trap RVT100-Savant). Gel pieces were rehydrated in 15 μL of 50 mM ammonium bicarbonate containing 20 ng/µL of sequencing grade modified trypsin. This step was performed for 40 min at 4°C and, after that, 20 μL of 50 mM ammonium bicarbonate was added to keep the gel pieces wet during tryptic digestion (37°C, 16 h). To extract peptides, 20 μL of 0.5% (v/v) trifluoroacetic acid (TFA) in 50% (v/v) ACN were added and samples were sonicated three times for 10 min each in a sonicator bath. The supernatant was recovered and concentrated under vacuum to a volume of approximately 10 μL. The resulting peptides were extracted, partially dried and salts were removed using C18 ZipPlate, following the manufacturer’s instructions. The tryptic peptides were analysed in a 4700-Proteomics Analyzer MALDI-TOF/TOF (Applied Biosystems, USA).^39^

*Database searching and criteria for protein identification -* The sequencing data were applied to the Mascot search engine (www.matrixscience.com) using the National Center for Biotechnology Information (NCBI) database, set to consider trypsin as the digestion enzyme and for peptide tolerance of +/21.2 Da. The following types of modifications were specified as variables for this study: acetyl (protein N-term), carbamidomethyl (C), deamidated (NQ), Gln RPyro-Glu (N-term-Q), Glu RPyro-Glu (N-term-E), oxidation (HW) and oxidation (M). Criteria for protein identification included Mascot scores, a sequence coverage > 50, concordance between predicted molecular mass and isoelectric point compatible with the values observed in the 2DE gel assays.

*Bioinformatic analyses -* The enzyme commission (EC) number, transmembrane domains, signal peptide and Gene Ontology (GO) terms were functional information on proteins obtained using The UniProt Knowledgebase (UniProtKB) (https://www.uniprot.org/). In addition, the proteins identified were analysed using VectorBase analysis tool release 48 (https://vectorbase.org/) for enrichment of GO and Metabolic Pathway annotations. The parameters used were organism *Lu. longipalpis* Jacobina with a p-value cut-off of 0.05.

*Selection of targets for analysis of gene expression -* The proteins observed in the proteomics assays, correctly identified by their gi/code (www.ncbi.nlm.nih.gov/pubmed) had their sequences of nucleic acids analysed for the design of specific PCR primers using the online software Primer3 v. 0.4.0 (http://frodo.wi.mit.edu/primer3/). Peptide sequences generated by MS/MS - MALDI-TOF/TOF were analysed by VectorBase (VB-2015-12) for the genome of *Lu. longipalpis*.

*Adhesion of Lulo cells-promastigotes in vitro -* Infective *L. (V.) braziliensis* promastigotes (strain MCAN/BR/1998/619), kindly provided by Dr Maria de Fátima Madeira (Evandro Chagas National Institute of Infectious Diseases, Oswaldo Cruz Foundation), were maintained at 27ºC as a stock culture in Novy, MacNeal and Nicolle medium and subcultured every four days. The promastigotes stock cultures were visualised daily and maintained for up three passages. Promastigote cultures were grown in Schneider’s medium supplemented with 10% heat inactivated FBS. Before being used in the assays, these cultures were washed twice in PBS by centrifugation (3,800 *g*, 10 min, 4°C).^29^ Lulo cells cultured in 25 cm^2^ flasks were used for interaction with *L. (V.) braziliensis* at 27ºC in biological triplicate assays. Two hours after co-incubation, non-adherent promastigotes were removed for two wash cycles with PBS. The cell-parasite (I) and cell without parasite (wI) interactions were added to Grace/L15 medium and the assays followed at the times (8, 14, 24 and 48 h).

*RNA and cDNA preparation -* The triplicate samples of Lulo cell before and after adhesion assays were washed with PBS and 1 mL of TRizol was added to the monolayers and were then subsequently removed mechanically using scrapers and stored at -70°C. Then, RNA was extracted according to manufacture’s recommendations. RNA samples were treated with DNase I, quantified in a Spectrophotometer Pico 200 (Picodrop Ltd., UK) and stored at -70^o^C until further use. cDNA for the qPCR assays was synthesised from 5 µg of total RNA using SuperScript III First-Strand Synthesis kit and, later, quantified using Qubit ssDNA Assay Kit, to be adjusted to a final concentration of 1 ng/µL.

*RT-qPCR assays -* The gene sequences were selected for expression analysis and corresponding primers were designed for this study (Table). For real-time PCR assays, 2 ng of cDNA were used in a final reaction volume of 15 µL, containing 7.5 µL of Power Green PCR Master Mix and 10 pMol (each) of forward and reverse primers. PCR were conducted in a thermocycler 7500 Fast Real Time PCR System (Applied Biosystem) in 96 well plates. PCR cycle conditions were as follows: initial step 95ºC/10 min (one cycle); amplification step 95ºC/30 s, 60ºC/30 s, 72ºC/30 s (40 cycles). A dissociation curve was performed after each assay to check the quality of each primer. Gene expression levels were calculated by relative quantitation using the comparative Ct method (^ΔΔ^Ct), as previously described,^39^ with a baseline set at 0.02. *Lu. longipalpis* GAPDH (GenBank: ACPB02038754) and RP 49 (GenBank: DQ207738) were used as reference genes after validation of housekeeping gene expression between the samples in this study.^40^ Gene expression analysis, as well the reference gene validation, were performed using Expression Suite Software (Life Technologies).

**TABLE Primers:** and standard curve parameters for qPCR assays

Gene target	Primer sequences	Reference	Amplicon length (bp)	Slope	Intercept	Coefficient of linearity (r^2^)	Amplification efficiency (%)
GAPDH	Fw 5’-TTCGCAGAAGACAGTGATGG-3’ Rv 5’-CCCTTCATCGGTCTGGACTA-3’	This study	132	-3.22	20.78	0.99	104.30
RP49	Fw 5’-GACCGATATGCCAAGCTAAAGCA-3’ Rv 5’-GGGGAGCATGTGGCGTGTCTT-3’	Tinoco-Nunes et al.^40^	135	-3.23	19.97	0.99	104.00
LLOJ000219	Fw 5’-GCAATTGCTTGTTGCTCAAA-3’ Rw 5’-ATGAACGTGTCCTCCGTTTC-3’	This study	133	-3.05	28.96	0.99	112.64
LLOJ000326	Fw 5’-CATGTCGGGTGGCAAGTATG-3’ Rv 3’-AGCCCCTTCAGTGTAGTGTC-3’	This study	172	-3.18	19.06	0.99	106.20
LLOJ006663	Fw 5’-TCCCATGAAGAAGGCACCAC-3’ Rv 5’-TTGCAGCTGGTAGTGCATCA-3’	This study	179	-3.55	23.30	0.99	91.16

*Statistical analysis -* Image data of three 2DE gels were analysed by Student’s *t* test and p-value ≤ 0.05 was considered statistically significant. The RT-qPCR assays were performed from kinetic triplicates and the results were expressed as mean ± standard deviation (SD) and normalised by Shapiro-Wilk test. The statistical tests of the ^Δ^Ct values (student *t* test or Mann Whitney *U* sum test and analysis of variance) were performed with the GraphPad prism software version 5.00 for Windows (GraphPad Software, USA).

## RESULTS AND DISCUSSION

The state-of-the-art on Lulo cells is part of a growing discussion on the use of this insect cell lineage in understanding complex interactions involving parasites and their sandfly hosts.^27^ ^-^ ^30^ In the present work, we present the proteomic profile of the Lulo cell line and include the expression pattern for some genes influenced by interaction with *Leishmania* promastigotes. Some of the cellular physiological changes induced by interaction with the parasite, at the early points of contact, are also discussed.

**Fig. 1:**
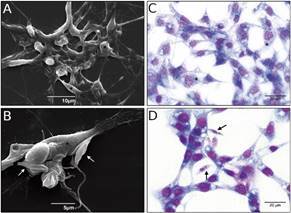
culture of *Lutzomyia longipalpis*-derived cell line (Lulo) monolayers. The cells were cultivated for 48 h until the cultures reached a semi-confluent monolayer, as observed by scanning electron microscopy (A and B) and Giemsa-stained light microscopy (C and D). Arrows indicate *Leishmania (Viannia) braziliensis* promastigotes adhered by the body or flagellum to Lulo cells (*).

**Fig. 2:**
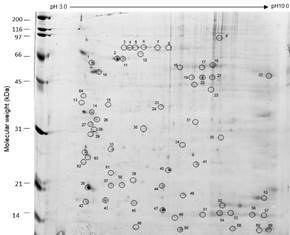
two-dimensional electrophoresis profile of *Lutzomyia longipalpis*-derived cell line (Lulo) protein extracts. The proteins were applied to gradient pH strips and subsequently resolved in 12% sodium dodecyl sulfate polyacrylamide gel electrophoresis (SDS-PAGE) gels stained by colloidal Coomassie Brilliant blue G-250. The 64 spots marked with circles and numbered were subsequently processed for matrix-assisted laser desorption and ionisation time-of-flight mass spectrometry (MALDI-TOF/TOF MS). The molecular weight of marker proteins is indicated in kDa. The gel figure is representative of three independent experiments.

In the first part of this study, we analysed the proteomic profile of Lulo cell monolayers (Fig. 1). Lulo cell cultures were processed and the whole cell extract was used in the proteomic assays. This strategy led to the observation of approximately 360 spot patches resolved in 2-DE, which allowed the resolution of proteins with molecular masses ranging from 12 to 97 kDa and a pI ranging from 4.0 to 9.5. A total of 64 of these points were identified by mass spectrometry, as indicated in the proteomic map (Fig. 2).

The proteomic results were further evaluated using Mascot MS/MS search engine and for each identified spot the following data was acquired: accession number, species, experimental isoelectric point (pI exp), experimental molecular weight (MW exp), peptides and the total score (Supplementary data, Table I). In order to confirm protein identification by the Mascot MS/MS server, all identified proteins sequences were then verified against the *Lu. longipalpis* genome, available in the VectorBase database (www.vectorbase.org) (Supplementary data, Table II). These analyses showed identity rates > 80% and coverages of 100% for all identified peptides.

**Fig. 3:**
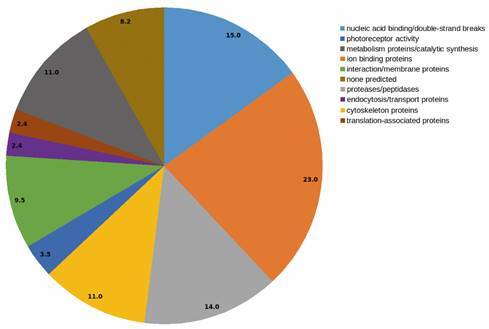
*in silico* analysis of the functional categories of the *Lutzomyia longipalpis*-derived cell line (Lulo) proteins identified in this study. Protein functions were defined using the UniProKB database (Protein Knowledgebase; http://www.uniprot.org/, up to date as of December 20, 2019). The data were processed by a personalised analysis based on the protein functions listed in the Supplementary data, Table II, and then they were grouped into eight categories and the percentage assigned (%) is represented in a pie chart.

Identified proteins were grouped into functional categories and included ion-binding proteins (23%), nucleic acid-binding proteins (15%), proteases/peptidases (14%), proteins related to metabolism/synthesis (11%), cytoskeleton proteins (11%), interaction/membrane proteins (9.5%), photoreceptor activity proteins (3.5%), translation-associated proteins (2.4%) and endocytosis/transport protein (2.4%). Some spots could not be classified: spots 7, 12, 13, 29, 33, 40 and 63 (totalling 8.2%) (Fig. 3). 

Although some data on insect proteins and/or their respective genes are available in the VectorBase database, the *Lu. longipalpis* genome is still incomplete and thus caused some gaps in the identification of proteins obtained in our study (those marked as “none predicted” in Supplementary data, Table II). However, it was possible to identify Lulo cell proteins with a single specific protein molecular function (n = 53 spots) or with two or more molecular functions (n = 11 spots).

The accession number (Mascott - Supplementary data, Table I) of some protein spots (1, 15, 43 and 46) could not be identified in the VectorBase genome *Lu. longipalpis* - LLOJ, but their functions were inferred in homology with other Diptera insects (Supplementary data, Table II). However, the protein spots 10, 14, 25, 27, 30, 31, 36, 38, 39, 44, 53, 55, 57, 58, 60, 62 and 64 were identified in the *Lu. longipalpis* genome - LLOJ, showing a structural similarity but, so far, these proteins have not been characterised by their functions.

In addition, the accuracy of the results shown here by homology analysis could be due to the fact that Phlebotominae are more closely related to *Anopheles*, a Diptera of the Nematocera suborder along with many bloodsucking insects (mosquitoes, black flies and biting “midges”).^41^

Additional bioinformatics analyses showed three proteins that are integral components of the membrane presented G-protein coupled receptor activity and seven predicted transmembrane domains (Supplementary data, Table II). In relation to N-terminal signal peptide, only three proteins are predicted by the tools in UniProtKB (Supplementary data, Table II).

The data analysis based on GO and Metabolic Pathway annotations of VectorBase showed that “Glycolysis/Gluconeogenesis” (9.59-fold) and “inositol phosphate metabolism” (5.62-fold) are overrepresented metabolic pathways (Supplementary data, Table III). The GO enrichment related to molecular function showed that “structural constituents of cytoskeleton” (105.14-fold), “translation elongation factor activity” (50.07-fold), “isomerase activity” (18.45-fold) and “ATP binding” (2.60-fold) are enriched GO terms (Supplementary data, Table III). In addition, the analysis related to biological process showed that “ATP generation from ADP” (43.81-fold), “glycolytic process” (43.81-fold), “translational elongation” (43.81-fold), “protein folding” (22.47-fold), “cytoskeleton organisation” (9.60-fold) and “microtubule-based process” (6.26-fold) are enriched GO terms (Supplementary data, Table III). In relation of cellular component, “nucleosome” (40.44-fold), “transport vesicle membrane” (25.03-fold), “microtubule” (18.77-fold) and “cytoskeleton” (7.36-fold) are overrepresented terms (Supplementary data, Table III). This enrichment analysis corroborates differences in energetic metabolism mainly related to glycolysis in the folding and translation of proteins, as well as changes in the cell’s cytoskeleton and transport of vesicles.

We evaluated the gene expression patterns for some genes in uninfected Lulo cells and compared them to the patterns arising from interaction with *L. (V.) braziliensis* promastigotes. Monolayers of Lulo cells, with their typical morphological features, such as epithelioid shape, were co-incubated with parasites and adhesion of promastigotes was registered both by scanning electron microscopy and optical microscopy (Fig. 1). These assays indicated that *L. (V.) braziliensis* promastigotes could adhere to and be internalised by Lulo cells after 8 h of interaction (Supplementary data, Video).

Among the 64 proteins identified by mass spectrometry in Lulo cell, three were elected for gene expression analysis after interaction with promastigotes: enolase (LLOJ000219), spot 19; tubulin (LLOJ000326), spot 17; and an unknown protein related to vacuolar transport and metal ion binding (LLOJ006663), spot 31. These proteins are associated with these biological processes and were selected based on their putative functions described in the *Lu. longipalpis* genome in VectorBase (Supplementary data, Table II), as well as their importance in the literature.^42^ ^,^ ^43^ In addition, identification with a score greater than 50 reflects the quality of the peptide sequences obtained and the consequent identification of the protein, in the studied condition. These three proteins had their corresponding nucleic acid sequences analysed for the design of specific RT-PCR primers (Table).

The comparison of gene expression patterns demanded standardisation of the PCR and validation of the relative e quantification by the ^ΔΔ^Ct method. Thus, the PCR efficiencies, determined by cDNA serial dilution curves, were compared (Supplementary data, Figure A). Moreover, melt curve analysis for all gene targets was performed to verify the specific amplification of the target genes, as represented by a single peak in each melt curve (Supplementary data, Figure B). The PCR efficiency for each target gene was assessed: 112.64% for LOJ000219 target; 106.2% for LLOJ000326 target; and 91.16% for LLOJ006663 target. For the reference genes, efficiencies were 104.3 and 104% for GAPDH and RP49, respectively (Table). No peaks were observed in the negative template control, indicating the absence of primer-dimers in the PCR. The LLOJ000219 presented an intermediary basal expression (Ct between 27 and 28), whereas LLOJ000326 and LOJ006663 presented a high basal expression (Cts between 20 and 25) (Supplementary data, Figure C). 

**Fig. 4:**
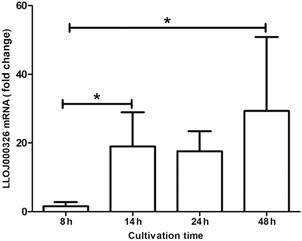
profile of gene expression of the β-tubulin gene in *Lutzomyia longipalpis*-derived cell line (Lulo) at multiple time points. The assays were performed at different times (8, 14, 24 and 48 h) post-seeding of the Lulo cell monolayers. The calibration curves 1:5 serial dilution of cDNAs from 60 ng. After cDNA synthesis, gene expressions were assessed by real-time quantitative PCR. The results are shown in fold change of mRNA in cDNA samples. The results represent the mean ± standard deviation of data from three biological and experimental assays. Asterisks indicate statistically significant differences (< 0.05) in fold change values when compared to data from the previous time point.

After PCR standardisation, gene expression profiles of control group Lulo cell monolayers were observed, using biological and experimental triplicates, at multiple time points: 8, 14, 24 and 48 h post-seeding, using the 8 h time-point as calibrator. Only for the gene LLOJ000326, a statistically significant difference (p < 0.05) was found at 14 h (19.02 ± 9.94) and 48 h (29.32 ± 21.55) (Fig. 4). 

**Fig. 5:**
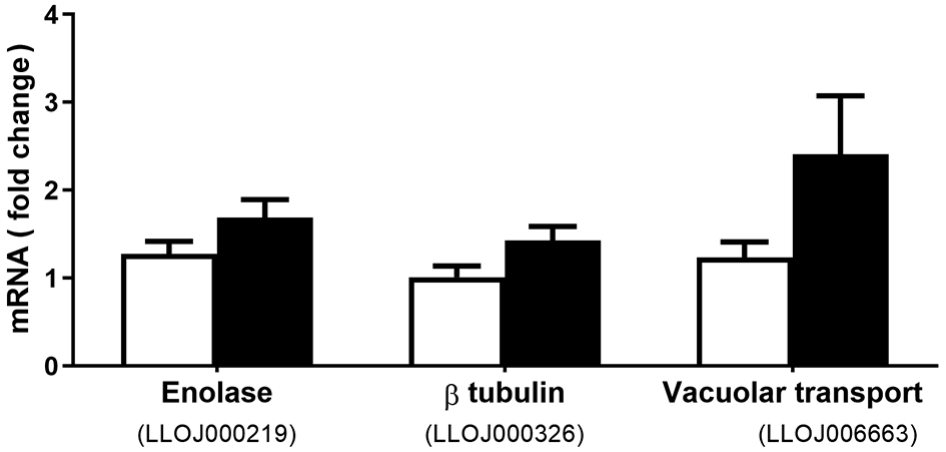
profile of gene expression of the enolase, β-tubulin and vacuolar transport protein genes in *Lutzomyia longipalpis*-derived cell line (Lulo). The assays were performed at 8 h post-seeding of the Lulo cell monolayers without (□) and with *Leishmania (Viannia) braziliensis* promastigotes (■). After cDNA synthesis, gene expressions were assessed by real-time quantitative PCR. The results are shown in fold-change of mRNA in cDNA samples of enolase (LLOJ000219), β-tubulin (LLOJ000326) and vacuolar transport protein (LLOJ006663). The results represent the mean ± standard deviation of data from three biological and experimental assays.

Looking at the *in vitro* study of the Lulo cell monolayer interaction with the parasite *L. (V.) braziliensi*s, the degradation occurs at 14, 24 and 48 h (data not shown) due to the strong adhesion of both surface proteins (Lulo cell - parasite), as shown in the video “multiple parasites invading a single cell” (Supplementary data, Video). The parasite seeks adaptation to the new environment, multiplying and causing degradation and imbalance of RNA, with this cell structures and genes can be activated or repressed. Nevertheless, 8 h after parasite interaction began, an increase in the expression of the three selected genes in Lulo cells was observed from the normalisation with the reference genes [gene LLOJ000219 (1.69 ± 0.55), gene LLOJ000326 (1.43 ± 039) and gene LOJ006663 (2.41 ± 1.88)], when compared to control Lulo cell monolayers without parasite interaction [gene LLOJ000219 (1.27 ± 0.41), gene LLOJ000326 (1.01 ± 0.36) and gene LOJ006663 (1.24 ± 0.49)] (Fig. 5). Although no statistical difference could be observed so far, a tendency of an increase in the expression of the three selected genes could be observed, especially in the vacuolar transport gene - LOJ006663 (Fig. 5).

The alteration in the expression of these genes when the parasite is in contact with the Lulo cells suggested that these cells are sensitive to physiological changes in the presence of the parasite, with possible changes in transcriptional activity. An interesting fact observed in this work is the 1.4-fold increase in the expression of the β-tubulin gene (LLOJ00326) (Fig. 4). The LLOJ00326 protein is a major structural constituent of microtubules, which form the cytoskeleton of mammalian cells. The β-tubulin forms dimer with alpha-tubulin to produce a typical microtubule, comprised of alpha-beta heterodimers.^44^ ^,^ ^45^ These data suggest that Lulo cells may be induced to express levels of β-tubulin in the first hours of interaction with the parasite (Fig. 5).

The gene encoding enolase (LLOJ000219) showed a 1.3-fold increase in gene expression within 8 h of interaction when compared to uninfected controls. Enolases are intracellular enzymes that have multiple functions and are considered a key glycolytic enzyme in the cytoplasm of cells, catalysing the interconversion of 2-phosphoglycerate to phosphoenolpyruvate. These enzymes form homodimers with magnesium ions non-covalently bound to the active site.^46^ ^)^ Enolases were described in the midgut transcriptome of *Lu. longipalpis* ^23^ ^)^ and it is suggested that they would have increased expression associated with the blood-feeding activity of the insect. As well as this, these enzymes were described as being involved in many processes of parasite and hematophagous insects, playing a role in the parasite interaction with the vector, acting on extracellular matrix degradation and/or preventing the coagulation of blood during the initial stage of parasite invasion.^23^

An interesting finding was the altered gene expression rates for vacuolar transport protein (LLOJ006663): 1.9-fold (Fig. 5). This protein is related to intracellular vacuolar membrane organisation and may possibly be related to the subversion of membrane transport pathways by vacuolar pathogens.^47^ An increased level of expression of this protein can induce certain biochemical functions related to the control of membrane transport in the host cell. It is important to emphasise that this finding corroborates the evidence that initiated interaction events, favouring the internalisation of the parasite, as shown in Supplementary data, Video.

The set of results presented here reinforce the intracellular function of proteins such as enolase, tubulin and vacuolar transport protein induced by the parasite-host interaction. This is the first report that describes *in vitro* the possibility of *Leishmania Viannia* sp., having an intracellular phase at a temperature of 27°C,^29^ although this may not reflect the interactions that occur in the parasite cycle inside the insect vector. 

In conclusion, the data presented here enrich the knowledge about Lulo epithelial cell line, which may be useful for the *in vitro* study of the surface components related to adhesion and can mimic the events that occur in the digestive tract of sandflies infected by *Leishmania* spp. This study is a pioneer in the field of Lulo cells, presenting the map of proteins that may be researched in further biochemical and molecular analyses.

The proteomic approach allowed for the identification of various Lulo cell proteins, according to functional groups: binding proteins, nucleic acid-binding proteins, proteases/peptidases, metabolism/synthesis proteins, cytoskeleton proteins, interaction/membrane proteins, photoreceptor activity proteins, translation associated proteins and endocytosis/transport protein. Such information may be useful for the further understanding of *Lu. longipalpis* physiology and its interactions with *Leishmania* spp.
