# Supplementary material for: Insights into the proteomic profile and gene expression of Lutzomyia longipalpis-derived Lulo cell line
Source: Mem Inst Oswaldo Cruz. 2020 Oct 26;115:e200113. doi: 10.1590/0074-02760200113 (PMC7586444; doi:10.1590/0074-02760200113)
Supplement: Supplementary file 1 [file 1678-8060-mioc-115-e200113-s.pdf]

TABLE I

Lutzomyia longipalpis-derived cell line (Lulo) proteins identified by MALDI-TOF/TOF 4700

Spot	Accession	Species[a]	pI teor	pI exp	MW teor	MW exp	Peptides number	Total score[b]
1	gi|157107430	*Aedes aegypti*	4.9	4.8	55.9	60.2	1	59
2	gi|58391242	*Anopheles gambiae*	5.5	5.4	60.7	60.2	2	102
3	gi|312384742	*Anopheles darlingi*	5.6	5.7	96.7	72.8	5	370
4	gi|195110283	*Drosophila mojavensis*	5.3	5.9	71.1	73.1	4	321
5	gi|195110283	*Drosophila mojavensis*	5.3	6.1	71.1	72.5	4	315
6	gi|194741132	*Drosophila ananassae*	6.3	6.3	69.8	72.8	1	85
7	gi|157130229	*Aedes aegypti*	4.5	6.5	42.8	71.2	4	346
8	gi|157665	*Drosophila melanogaster*	5.3	7.1	71.0	71.4	2	162
9	gi|195171061	*Drosophila persimilis*	6.2	7.4	61.0	86.7	1	79
10	gi|195452246	*Drosophila persimilis*	4.6	4.8	57.2	50.1	2	50
11	gi|58391242	*Anopheles gambiae*	5.5	5.5	60.7	60.2	2	102
12	gi|157116872	*Aedes aegypti*	5.3	6.2	69.7	63.9	1	73
13	gi|157130229	*Aedes aegypti*	4.5	4.0	23.2	38.2	4	346
14	gi|157138290	*Aedes aegypti*	4.6	4.6	49.3	36.0	1	50
15	gi|94468924	*Aedes aegypti*	5.2	4.7	32.5	38.3	1	79
16	gi|111035020	*Aedes aegypti*	5.0	5.9	44.5	55.8	4	232
17	gi|158749	*Drosophila melanogaster*	6.0	6.6	50.8	56.8	4	67
18	gi|195062513	*Drosophila grimshawi*	6.1	7.0	50.6	57.2	3	87
19	gi|157674465	*Lutzomyia longipalpis*	6.2	6.2	46.7	48.7	4	328
20	gi|157674465	*Lutzomyia longipalpis*	6.7	6.7	46.7	48.9	8	707
21	gi|157674465	*Lutzomyia longipalpis*	7.2	7.2	46.7	49.1	2	164
22	gi|7915	*Drosophila melanogaster*	8.8	9.5	50.2	46.7	1	80
23	gi|156773	*Drosophila melanogaster*	7.0	7.0	41.7	41.1	3	278
24	gi|157674465	*Lutzomyia longipalpis*	6.3	6.3	46.7	44.7	8	709
25	gi|312373340	*Anopheles darlingi*	6.5	5.5	43.1	37.6	1	84
26	gi|158293252	*Anopheles gambiae*	4.8	4.6	24.7	33.9	1	84
27	gi|289741489	*Glossina morsitans*	4.5	4.5	29.4	33.0	6	386
28	gi|158700	*Drosophila melanogaster*	4.8	4.6	29.3	31.6	5	285
29	gi|157114199	*Aedes aegypti*	4.3	4.3	21.7	28.0	1	85
30	gi|157122974	*Aedes aegypti*	5.3	5.2	29.8	30.4	2	156
31	gi|157134556	*Aedes aegypti*	6.2	6.1	22.4	32.2	2	99
32	gi|17352457	*Drosophila melanogaster*	4.7	4.6	23.9	26.5	1	81
33	gi|157114199	*Aedes aegypti*	5.3	4.7	21.7	27.4	1	85
34	gi|204305171	*Lutzomyia longipalpis*	5.8	5.8	17.2	27.7	5	354
35	gi|195055326	*Drosophila grimshawi*	8.1	7.5	28.2	29.0	2	130
36	gi|126507667	*Philoliche rondani*	5.8	4.5	34.0	21.5	1	160
37	gi|118779554	*Anopheles gambiae*	5.1	4.7	17.6	20.4	1	71
38	gi|1168731	*Drosophila melanogaster*	6.7	4.9	17.1	20.5	1	67
39	gi|195028448	*Drosophila grimshawi*	5.8	5.1	46.1	21.1	2	115
40	gi|110671518	*Diaphorina citri*	5.6	5.6	19.4	23.7	3	247
41	gi|157361521	*Phlebotomus papatasi*	6.3	6.3	21.9	24.3	4	274
42	gi|195340406	*Drosophila sechellia*	4.5	4.5	20.1	17.0	1	58
43	gi|17137634	*Drosophila melanogaster*	5.0	4.6	10.1	16.9	1	67
44	gi|17136986	*Drosophila melanogaster*	6.7	5.5	17.3	20.3	3	191
45	gi|296937154	*Phlebotomus papatasi*	6.0	5.1	15.3	16.2	2	170
46	gi|157361539	*Phlebotomus papatasi*	8.0	5.8	19.5	17.9	2	147
47	gi|296937154	*Phlebotomus papatasi*	6.0	5.5	15.3	15.0	6	433
48	gi|158298819	*Anopheles gambiae*	5.6	5.1	13.6	13.8	2	134
49	gi|195356624	*Drosophila sechellia*	6.4	6.4	13.3	13.9	1	52
50	gi|157117168	*Aedes aegypti*	7.0	5.7	11.5	12.4	3	236
51	gi|7436	*Drosophila hydei*	6.2	6.2	13.7	14.0	2	146
52	gi|157361595	*Maconellicoccus hirsutus*	6.5	6.5	13.4	15.4	1	78
53	gi|121543595	*Maconellicoccus hirsutus*	8.9	9.6	17.9	16.3	1	53
54	gi|24585671	*Drosophila melanogaster*	6.8	6.8	13.6	14.0	5	297
55	gi|58585202	*Apis mellifera*	7.4	7.4	15.0	14.0	1	71
56	gi|195356624	*Drosophila sechellia*	8.9	8.9	13.3	14.3	1	52
57	gi|157112948	*Aedes aegypti*	9.8	9.5	15.7	14.1	1	84
58	gi|157111905	*Aedes aegypti*	7.3	7.3	14.7	12.3	2	124
59	gi|195356624	*Drosophila sechellia*	8.1	8.1	13.3	12.3	1	52
60	gi|157169523	*Aedes aegypti*	9.0	10.7	13.3	13.3	1	67
61	gi|195356624	*Drosophila sechellia*	5.7	5.7	22.3	21.3	1	52
62	gi|21483444	*Drosophila melanogaster*	4.5	4.5	25.0	25.0	1	52
63	gi|194858178	*Drosophila erecta*	4.7	4.6	26.5	26.5	4	105
64	gi|195570334	*Drosophila simulans*	5.6	4.6	50.1	39.1	3	50

The extensive homology with insect species (*a*) indicate significant identity score > 50(*b*). MW exp: experimental molecular weight; MW teor: teorical molecular weight; pI exp: experimental isoelectric point; pI teor: teorical isoelectric point.

CC BY

TABLE II
Lutzomyia longipalpis-derived cell line (Lulo) proteins accessed in VectorBase by sequence homology analysis and functional analysis

Spot	*Lu. longipalpis* genome	Protein name	UniProtKB	Molecular function	Biological process	Cellular component	EC number	TM domains[a]	SignalP[b]
1	AM098860.1	AAELOO3035-PA	Q0IGC7	G-protein coupled receptor activity; photoreceptor activity	Phototransduction; protein-chromophore linkage; visual perception	Integral component of membrane	None predicted	7	None predicted
2	LLOJ009985	T-complex protein 1 subunit gamma	A0A1B0GLJ4	ATP binding; unfolded protein binding	Protein folding; regulation of apoptotic process	Cytoplasm	None predicted	None predicted	None predicted
	LLOJ000479	None predicted protein	A0A1B0C957	Nucleic acid binding	None predicted	None predicted	None predicted	None predicted	None predicted
3	LLOJ001213	Heat shock protein 70 cognate 2	A0A1B0GHD1	ATP binding	None predicted	None predicted	None predicted	None predicted	None predicted
4	LLOJ001213	Heat shock protein 70 cognate 2	A0A1B0GHD1	ATP binding	None predicted	None predicted	None predicted	None predicted	None predicted
5	LLOJ001213	Heat shock protein 70 cognate 2	A0A1B0GHD1	ATP binding	None predicted	None predicted	None predicted	None predicted	None predicted
6	LLOJ008388	Heat shock protein	A0A1B0GK60	ATP binding	None predicted	None predicted	None predicted	None predicted	None predicted
7	LLOJ003036	None predicted protein	A0A1B0CFB5	None predicted	None predicted	Nascent polypeptide-associated complex	None predicted	None predicted	None predicted
8	LLOJ008388	Heat shock protein	A0A1B0GK60	ATP binding	None predicted	None predicted	None predicted	None predicted	None predicted
	LLOJ005733	Heat shock protein 70 Ba	A0A1B0CM45	ATP binding	None predicted	None predicted	None predicted	None predicted	None predicted
9	LLOJ003520	Mitochondrial heat shock protein	A0A1B0GI32	ATP binding; unfolded protein binding	Protein folding	None predicted	None predicted	None predicted	None predicted
10	LLOJ000164	None predicted protein	A0A1B0C8F2	G-protein coupled receptor activity	None predicted	Integral component of membrane	None predicted	7	None predicted
11	LLOJ009985	T-complex protein 1 subunit gamma	A0A1B0GLJ4	ATP binding; unfolded protein binding	Protein folding; regulation of apoptotic process	Cytoplasm	None predicted	None predicted	None predicted
	LLOJ000479	None predicted protein	A0A1B0C957	Nucleic acid binding	None predicted	None predicted	None predicted	None predicted	None predicted
12	LLOJ004651	None predicted protein	A0A1B0CJE9	None predicted	None predicted	None predicted	None predicted	None predicted	Yes
13	LLOJ003036	None predicted protein	A0A1B0CFB5	None predicted	None predicted	Nascent polypeptide-associated complex	None predicted	None predicted	None predicted
14	LLOJ000532	None predicted protein	A0A1B0C9B0	Nucleic acid binding	None predicted	None predicted	None predicted	None predicted	None predicted
15	AAEL003035	Putative opsin 2	A0A0P6J590	G-protein coupled receptor activity; photoreceptor activity	Phototransduction; protein-chromophore linkage; visual perception	Integral component of membrane	None predicted	7	None predicted
16	LLOJ000326	Tubulin beta chain	A0A1B0C8Q4	GTPase activity; GTP binding; structural constituent of cytoskeleton	Microtubule-based process	Microtubule	None predicted	None predicted	None predicted
	LLOJ007632	None predicted protein	A0A1B0CRY4	GTPase activity; GTP binding; structural constituent of cytoskeleton	Microtubule-based process	None predicted	None predicted	None predicted	None predicted
17	LLOJ000326	Tubulin beta chain	A0A1B0C8Q4	GTPase activity; GTP binding; structural constituent of cytoskeleton	Microtubule-based process	Microtubule	None predicted	None predicted	None predicted
18	LLOJ000130	Tubulin beta chain	A0A1B0C8B8	GTPase activity; GTP binding; structural constituent of cytoskeleton	Microtubule-based process	Cytoplasm; microtubule	None predicted	None predicted	None predicted
19	LLOJ000219	Enolase	A8CWB5	Magnesium ion binding; phosphopyruvate hydratase activity	Glycolytic process	Phosphopyruvate hydratase complex	4.2.1.11	None predicted	None predicted
20	LLOJ000219	Enolase	A8CWB5	Magnesium ion binding; phosphopyruvate hydratase activity	Glycolytic process	Phosphopyruvate hydratase complex	4.2.1.11	None predicted	None predicted
21	LLOJ000219	Enolase	A8CWB5	Magnesium ion binding; phosphopyruvate hydratase activity	Glycolytic process	Phosphopyruvate hydratase complex	4.2.1.11	None predicted	None predicted
22	LLOJ008981	Putative GEF	A0A1B0CVJ5	GTPase activity; GTP binding; translation elongation factor activity	None predicted	None predicted	None predicted	None predicted	None predicted
23	LLOJ004800	Putative actin	A8CW58	ATP binding	None predicted	Cytoplasm; cytoskeleton	None predicted	None predicted	None predicted
24	LLOJ000219	Enolase	A8CWB5	Magnesium ion binding; phosphopyruvate hydratase activity	Glycolytic process	Phosphopyruvate hydratase complex	4.2.1.11	None predicted	None predicted
	LLOJ000221	None predicted protein	A0A1B0GGU9	None predicted	tRNA wobble uridine modification	Elongator holoenzyme complex	None predicted	None predicted	None predicted
25	LLOJ007043	None predicted protein	A0A1B0CQ94	RNA binding	None predicted	None predicted	None predicted	None predicted	None predicted

Spot	*Lu. longipalpis* genome	Protein name	UniProtKB	Molecular function	Biological process	Cellular component	EC number	TM domains[a]	SignalP[b]
26	LLOJ009318	Clathrin light chain	A0A1B0CWD4	Structural molecule activity	Intracellular protein transport; vesicle-mediated transport	Clathrin coat of coated pit; clathrin coat of trans-Golgi network vesicle	None predicted	None predicted	None predicted
27	LLOJ000837	None predicted protein	A0A1B0CA65	None predicted	None predicted	None predicted	None predicted	None predicted	None predicted
	AM094123.1	AAEL003035	Q0IGC7	G-protein coupled receptor activity; photoreceptor activity	Phototransduction; protein-chromophore linkage; visual perception	Integral component of membrane	None predicted	7	None predicted
	AM109782.1	Tropomyosin invertebrate	A0A6I8T7F4	None predicted	None predicted	None predicted	None predicted	None predicted	None predicted
28	LLOJ000837	None predicted protein	A0A1B0CA65	None predicted	None predicted	None predicted	None predicted	None predicted	None predicted
	AM094120.1	AAEL003035	Q0IGC7	G-protein coupled receptor activity; photoreceptor activity	Phototransduction; protein-chromophore linkage; visual perception	Integral component of membrane	None predicted	7	None predicted
	AM109782.1	Tropomyosin invertebrate	A0A6I8T7F4	None predicted	None predicted	None predicted	None predicted	None predicted	None predicted
29	LLOJ009638	None predicted protein	A0A1B0CXA2	None predicted	None predicted	None predicted	None predicted	None predicted	None predicted
30	LLOJ000338	None predicted protein	A0A1B0C8R6	ATP binding; nucleic acid binding	None predicted	None predicted	3.6.4.13	None predicted	None predicted
31	LLOJ006662	None predicted protein	A0A1B0GJM9	Metal ion binding	None predicted	Nucleolus	None predicted	None predicted	None predicted
	LLOJ006663	None predicted protein	A0A1B0GJN0	None predicted	Vacuolar transport	None predicted	None predicted	None predicted	None predicted
32	LLOJ006908	Peptidylprolyl isomerase	A0A1B0CPV9	Calcium ion binding; peptidyl-prolyl cis-trans isomerase activity	None predicted	None predicted	5.2.1.8	None predicted	None predicted
33	LLOJ009638	None predicted protein	A0A1B0CXA2	None predicted	None predicted	None predicted	None predicted	None predicted	None predicted
34	LLOJ004308	Triosephosphate isomerase	A0A1B0ETY1	Triose-phosphate isomerase activity	Gluconeogenesis; glycolytic process	None predicted	5.3.1.1	None predicted	None predicted
35	LLOJ005433	Phosphoglycerate mutase	A0A1B0CLE6	Bisphosphoglycerate mutase activity; phosphoglycerate mutase activity	Glycolytic process	None predicted	5.4.2.11; 5.4.2.4	None predicted	None predicted
36	LLOJ009278	Aspartate carbamoyltransferase	A0A1B0CW94	Amino acid binding; aspartate carbamoyltransferase activity; ATP binding; metal ion binding	'de novo' pyrimidine nucleobase biosynthetic process; glutamine metabolic process	None predicted	2.1.3.2; 3.5.2.3 6.3.5.5	None predicted	None predicted
37	LLOJ007340	None predicted protein	A0A1B0CR41	Ribosome binding; translation elongation factor activity	Positive regulation of translational elongation; positive regulation of translational termination	None predicted	None predicted	None predicted	None predicted
38	LLOJ002211	None predicted protein	A0A1B0CCZ1	Actin binding	Actin filament depolymerisation	Actin cytoskeleton	None predicted	None predicted	Yes
39	LLOJ002211	None predicted protein	A0A1B0CCZ1	Actin binding	Actin filament depolymerisation	Actin cytoskeleton	None predicted	None predicted	Yes
40	LLOJ001890	Transcription factor BTF3	A0A1B0GHX5	None predicted.	None predicted	None predicted	None predicted	None predicted	None predicted
41	LLOJ007174	Peptidyl-prolyl cis-trans isomerase	A0A1B0CQM5	Peptidyl-prolyl cis-trans isomerase activity	Protein folding	None predicted	5.2.1.8	None predicted	Yes
42	LLOJ008257	Transcription elongation factor spt6	A0A1B0CTQ7	DNA binding	Nucleobase-containing compound metabolic process; positive regulation of transcription elongation from RNA polymerase II promoter	Nucleus	None predicted	None predicted	None predicted
43	AM095132.1	Putative opsin 2	A0A0P6J590	G-protein coupled receptor activity; photoreceptor activity	Phototransduction; protein-chromophore linkage; visual perception	Integral component of membrane	None predicted	7	None predicted
44	LLOJ002211	None predicted protein	A0A1B0CCZ1	Actin binding	Actin filament depolymerisation	Actin cytoskeleton	None predicted	None predicted	Yes
45	LLOJ008594	Superoxide dismutase [Cu-Zn]	A0A1B0CUF9	Metal ion binding; superoxide dismutase activity	None predicted	None predicted	1.15.1.1	None predicted	None predicted
46	AM102059.1	Putative opsin 2	A0A0P6J590	G-protein coupled receptor activity; photoreceptor activity	Phototransduction; protein-chromophore linkage; visual perception	Integral component of membrane	None predicted	7	None predicted
47	LLOJ008594	Superoxide dismutase [Cu-Zn]	A0A1B0CUF9	Metal ion binding; superoxide dismutase activity	None predicted	None predicted	None predicted	None predicted	None predicted
	AM102059.1	Putative opsin 2	A0A0P6J590	G-protein coupled receptor activity; photoreceptor activity	Phototransduction; protein-chromophore linkage; visual perception	Integral component of membrane	None predicted	None predicted	None predicted
48	AM094299.1	Putative opsin 2	A0A0P6J590	G-protein coupled receptor activity; photoreceptor activity	Phototransduction; protein-chromophore linkage; visual perception	Integral component of membrane	None predicted	7	None predicted
	LLOJ006559	Profilin	A0A1B0CP86	Actin binding	None predicted	None predicted	None predicted	None predicted	None predicted

Spot	*Lu. longipalpis* genome	Protein name	UniProtKB	Molecular function	Biological process	Cellular component	EC number	TM domains[a]	SignalP[b]
49	LLOJ005281	Histone H2A	A0A1B0CKZ4	DNA binding; protein heterodimerisation activity	None predicted	Nucleosome; nucleus	None predicted	None predicted	None predicted
50	LLOJ003617	Peptidylprolyl isomerase	A0A1B0CGQ4	Peptidyl-prolyl cis-trans isomerase activity	None predicted	None predicted	5.2.1.8	None predicted	None predicted
51	LLOJ000528	Histone H2B	A0A1B0C9A6	DNA binding; protein heterodimerisation activity	Nucleosome assembly	Host cell nucleus; nucleosome; nucleus	None predicted	None predicted	None predicted
52	LLOJ000995	40S ribosomal protein S20-like protein	A8CWE6	Structural constituent of ribosome	Translation	Small ribosomal subunit	None predicted	None predicted	None predicted
53	LLOJ000748	None predicted protein	A0A1B0C9X6	peptidyl-prolyl cis-trans isomerase activity	Protein folding	None predicted	None predicted	None predicted	None predicted
54	LLOJ002917	Histone H2B	A0A1B0CEZ7	DNA binding; protein heterodimerisation activity	None predicted	Nucleosome; nucleus	None predicted	None predicted	None predicted
55	LLOJ009052	None predicted protein	A0A1B0EX54	Lipid binding	None predicted	None predicted	None predicted	None predicted	None predicted
56	LLOJ005281	Histine H2A	A0A1B0CKZ4	DNA binding; protein heterodimerisation activity	None predicted	Nucleosome; nucleus	None predicted	None predicted	None predicted
57	LLOJ009251	None predicted protein	A0A1B0CW67	Single-stranded DNA binding	DNA replication	None predicted	None predicted	None predicted	None predicted
58	LLOJ009052	None predicted protein	A0A1B0EX54	Lipid binding	None predicted	None predicted	None predicted	None predicted	None predicted
59	LLOJ005281	Histine H2A	A0A1B0CKZ4	DNA binding; protein heterodimerisation activity	None predicted	Nucleosome; nucleus	None predicted	None predicted	None predicted
60	LLOJ008701	None predicted protein	A0A1B0CUR6	ATP binding	Protein folding	None predicted	None predicted	None predicted	None predicted
61	LLOJ005281	Histone H2A	A0A1B0CKZ4	DNA binding; protein heterodimerisation activity	None predicted	Nucleosome; nucleus	None predicted	None predicted	None predicted
62	LLOJ001874	None predicted protein	A0A1B0CC94	Double-strand break repair via homologous recombination	None predicted	None predicted	None predicted	None predicted	None predicted
63	LLOJ007900	None predicted protein	A0A1B0CSQ1	None predicted	None predicted	None predicted	None predicted	None predicted	None predicted
64	LLOJ004788	None predicted protein	A0A1B0GIL9	Catalytic activity	None predicted	None predicted	None predicted	None predicted	None predicted

a: number of transmembrane domains predicted for the protein based in UniProtKB database; *b*: prediction of presence of signal peptides based in UniProtKB database.

TABLE III

Gene Ontology (GO) and Metabolic Pathway enrichment of *Lutzomyia longipalpis* proteins based in VectorBase database

GO ID	GO term	Genes in the bkgd[a] with this term	Genes in your result with this term	Percent of bkgd[a] genes in your result	Fold enrichment	Odds ratio	p-value	Benjamini	Bonferroni
Molecular function									
0016853	Isomerase activity	57	6	10.5	18.45	23.66	7.03e-7	6.47e-5	6.47e-5
0005200	Structural constituent of cytoskeleton	5	3	60.0	105.14	280.88	1.72e-6	7.70e-5	1.58e-4
0003746	Translation elongation factor activity	7	2	28.6	50.07	73.04	6.56e-4	4.93e-3	6.04e-2
0005524	ATP binding	606	9	1.5	2.60	3.06	6.26e-3	1.96e-2	5.76e-1
Biological process									
0006457	Protein folding	39	5	12.8	22.47	28.86	2.38e-6	4.45e-4	7.60e-4
0006757	ATP generation from ADP	12	3	25.0	43.81	62.36	3.68e-5	1.85e-3	1.18e-2
0006096	Glycolytic process	12	3	25.0	43.81	62.36	3.68e-5	1.85e-3	1.18e-2
0007010	Cytoskeleton organization	73	4	5.5	9.60	11.03	7.52e-4	7.65e-3	2.41e-1
0006414	Translational elongation	8	2	25.0	43.81	60.86	8.72e-4	8.14e-3	2.79e-1
0007017	Microtubule-based process	84	3	3.6	6.26	6.86	1.20e-2	4.26e-2	1.00e+0
Cellular component									
0000786	Nucleosome	13	3	23.1	40.44	56.12	4.76e-5	1.03e-3	4.14e-3
0005874	Microtubule	28	3	10.7	18.77	22.40	5.13e-4	5.51e-3	4.47e-2
0005856	Cytoskeleton	119	5	4.2	7.36	8.52	5.38e-4	5.51e-3	4.68e-2
0030658	Transport vesicle membrane	7	1	14.3	25.03	29.71	3.93e-2	1.01e-1	1.00e+0
KEGG pathways									
ec00010	Glycolysis /Gluconeogenesis	58	3	5.2	9.59	11.66	3.43e-3	5.82e-2	5.82e-2
ec00562	Inositol phosphate metabolism	66	2	3.0	5.62	6.29	4.82e-2	2.36e-1	8.20e-1

a: background (bkgd).

VIDEO
click here to access

Lutzomyia longipalpis-derived cell line (Lulo) monolayer with 8 h of the adhesion with promastigotes *Leishmania (Viannia) braziliensis.*

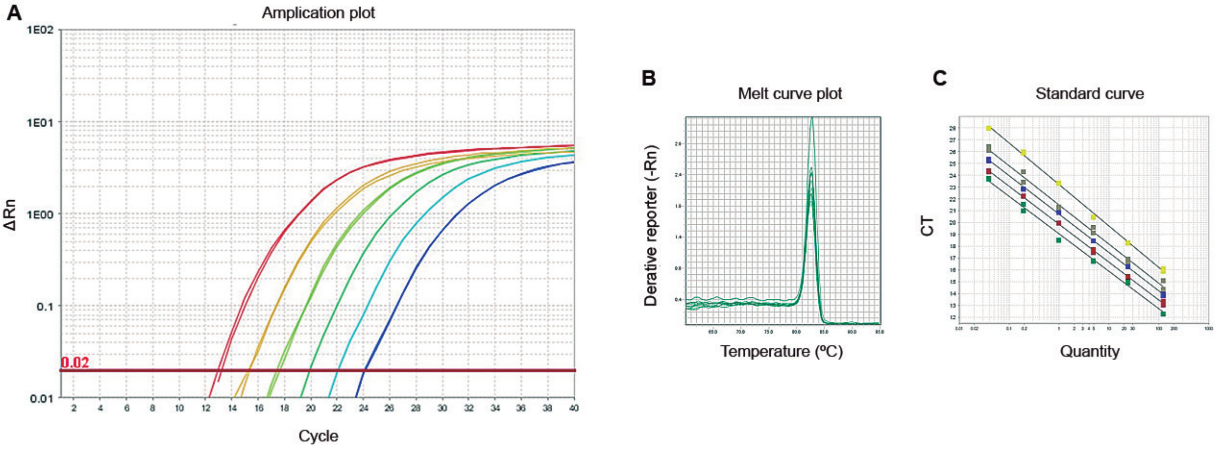

Figure: standardisation of qPCR assays for gene expression analysis in monolayers of *Lutzomyia longipalpis*-derived cell line (Lulo). (A) Representative amplification plot with the fluorescent signal magnitude. (B) Representative melting curve indicating the reaction specificity, observed by a single peak in each primer curve. (C) Calibration curve for the target sequences LLOJ000219 (yellow), LLOJ000326 (green), LLOJ006663 (grey), GAPDH (blue) and RP49 (red) from the genome of *Lu. longipalpis*. The efficiency of the PCR assays was assessed with calibration curves of serial dilution of 1: 5 cDNAs (60 ng). Gene expression levels were determined by the $^{\Delta\Delta}$Ct methodology (relative expression).
